# Axial Length as a Key Risk Factor for Lattice Degeneration: A Large-Sample Retrospective Analysis

**DOI:** 10.3390/healthcare14050561

**Published:** 2026-02-24

**Authors:** Qiulin Mi, Youruo Zhang, Yingying Nie, Xiaoxiao Wu, Haobo Fan, Mingxu Zhang, Junguo Duan

**Affiliations:** 1 School of Ophthalmology, Chengdu University of Traditional Chinese Medicine, Chengdu 610075, China; miqlins@163.com (Q.M.); zhangyouruo@cdzyydx1.wecom.work (Y.Z.); opthal66@cdutcm.edu.cn (Y.N.); wxx199701@163.com (X.W.); fanhaobo@cdzyydx1.wecom.work (H.F.); 2 Ineye Hospital of Chengdu University of Traditional Chinese Medicine, Chengdu 610084, China; 3 Eye Health with Traditional Chinese Medicine Key Laboratory of Sichuan Province, Chengdu 610075, China

**Keywords:** lattice degeneration, retinal detachment, ocular biometric parameters, risk prediction

## Abstract

Background: Lattice degeneration (LD) is a well-established precursor lesion of retinal detachment, a condition with often poor prognosis that can lead to permanent vision loss. Early identification of LD is clinically significant for risk assessment. This study aimed to investigate the associations between LD and ocular biometric as well as demographic parameters, and to develop a risk assessment framework. The ultimate goal is to identify individuals at genuinely high risk who may benefit from tailored follow-up or treatment. Methods: In this cross-sectional study, 1776 subjects from 7634 screened individuals at the Eye Health Management Center of Ineye Hospital, Chengdu University of Traditional Chinese Medicine, were enrolled. Comprehensive ophthalmic examinations were conducted. Chi-square tests compared LD prevalence across groups. Independent risk factors were identified through univariate and multivariate logistic regression analyses. Results: The overall prevalence of LD was 13.3%, with unilateral cases (9.3%) exceeding bilateral cases (4.0%). Prevalence was significantly higher in myopic (15.5%) versus non-myopic individuals (10.5%, *p* < 0.05), but did not differ by gender or ocular dominance. Spherical equivalent (SE), axial length (AL), axial length/corneal radius ratio (AL/CR), and age were positively correlated with LD risk (all *p* < 0.05). Age, AL, and best-corrected visual acuity (BCVA) were independently associated with the presence of LD. Among those AL may be a more directly relevant biological parameter for assessing LD risk, each 1.00 mm increase in AL increased risk by 53% (OR = 1.53). Conclusions: The overall prevalence of LD was 13.3%. Given that AL is a key independent risk factor for LD, it is recommended that individuals with elongated AL undergo peripheral retinal screening. This facilitates early detection and enables prophylactic treatment for those at high risk of retinal detachment.

## 1. Introduction

Lattice degeneration (LD) is a common peripheral retinal degenerative condition characterized by localized retinal thinning and weakening, often presenting with a lattice-like pattern. This structural alteration predisposes the area to retinal breaks and detachment. Optical coherence tomography (OCT) imaging frequently reveals vitreoretinal traction at the lesion site [1]. Although often asymptomatic itself, lattice degeneration is a significant clinical concern as a key risk factor for retinal detachment (RD) [2], with studies indicating its association in approximately 30% of RRD cases [3]. Retinal detachment can lead to severe and irreversible vision loss, underscoring the critical importance of early identification and management of lattice degeneration [4].

The epidemiological profile of lattice degeneration remains a subject of debate, as reported prevalence rates vary considerably across studies due to differences in surveyed populations, examination methodologies, and diagnostic criteria [5]. Some estimates suggest that lattice degeneration is present in about 8% of the general population, with approximately 7% of individuals over 40 years of age potentially having asymptomatic retinal breaks [3]. Its precise pathophysiological mechanism is not fully elucidated, with current theories predominantly involving vitreous traction [6], retinal ischemia [5], developmental anomalies [6], and genetic factors [7].

A growing number of studies suggest that myopia is associated with LD [8,9,10,11]. This link is of particular significance against the backdrop of a continuously rising global prevalence of myopia. It is projected that by 2050, the global myopic population will reach approximately 4.758 billion, constituting nearly half of the world’s population [12]. The situation is especially severe among Chinese adolescents, with an overall myopia rate of 52.7% in 2020, soaring to 80.5% among high-school students [12,13]. Within this trend, the prevention and control of myopia-related fundus pathologies gain heightened importance. The detection rate of lattice degeneration is significantly elevated in myopic populations, reported to be as high as 15% in myopic adolescents [14]. Axial elongation in myopia mechanically stretches the retina, substantially increasing the risk of lattice degeneration [15], which in turn elevates the risk of vision-threatening complications such as retinal detachment and maculopathy [16,17]. This not only causes personal visual impairment but also imposes a substantial socio-economic burden [16,18]. Therefore, in the context of the myopia epidemic, enhancing early screening for lattice degeneration carries significant public health implications.

A comprehensive assessment of the retinal periphery is crucial for diagnosing lattice degeneration. However, traditional examination methods are limited by restricted fields of view and patient discomfort [19]. Advances in imaging technology, such as ultra-widefield scanning laser ophthalmoscopy, enable rapid, non-invasive acquisition of 200° field-of-view fundus images, significantly improving the detection capability for peripheral lesions [20]. This technological progress provides a new platform for epidemiological and etiological research on lattice degeneration, making precise large-scale population screening feasible [21].

Based on this rationale, the present study utilizes ultra-widefield imaging to systematically screen for lattice degeneration. It comprehensively analyzes the correlation between the condition and key Ocular Parameters, including spherical equivalent (SE), mean keratometry (Km), axial length (AL), In previous studies on myopia, the axial length to corneal radius ratio (AL/CR) has been regarded as a composite parameter superior to AL alone. Therefore, we included AL/CR in our correlation analysis [22]. By identifying independent risk factors for LD, this study aims to facilitate early detection of high-risk individuals, thereby providing a basis for necessary interventions and ultimately reducing the risk of progression to severe complications such as retinal detachment.

## 2. Methods

### 2.1. Study Design and Participants

Considering that lattice degeneration (LD) is a retinal degenerative disease that occurs across all age groups and shows an age-related increase in prevalence, this cross-sectional study enrolled individuals aged 3 to 80 years who underwent ocular health examinations at the Eye Health Management Center of the Ineye Hospital of Chengdu University of Traditional Chinese Medicine (hereinafter referred to as the Ineye Hospital) between January 2019 and September 2025. Participants with a history of retinopathy of prematurity, Stickler syndrome, ocular trauma, uveitis, or orthokeratology treatment, as well as those with ungradable fundus image quality, were excluded. The study adhered to the ethical principles of the Declaration of Helsinki. The study protocol was approved by the Institutional Review Board of Ineye Hospital (Approval No. 2025YH003 on 27 February 2025). From an initial pool of 7634 screened individuals, following a comprehensive quality assessment, which involved the exclusion of participants due to reasons such as ungradable image quality, incomplete clinical data, or non-compliance with the inclusion criteria, a final cohort of 1776 participants was included in the analysis. For the analysis of age-specific prevalence, participants were categorized into age groups. Minors were defined as individuals aged below 18 years, based on standard demographic classifications. This grouping was used to calculate the prevalence of lattice degeneration in minors, as reported in the Section 3.

### 2.2. Ophthalmic Examinations

**Figure 1:**
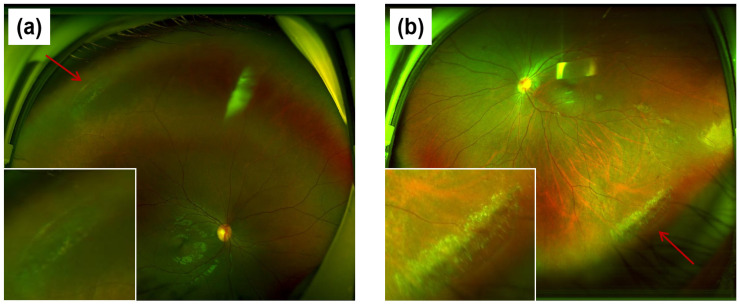
Images of peripheral fundus examination. (**a**,**b**) are images of the superior peripheral retina of the right eye and the inferior peripheral retina of the left eye, respectively. The area indicated by the arrow is the lattice-like retinal degeneration area, and the white box contains a magnified image.

Participants received a systematic ophthalmic examination, which included: measurement of best-corrected visual acuity (BCVA) using a standard international visual acuity chart; intraocular pressure (IOP) measurement and objective refraction using a fully automated autorefractor/keratometer (TONOREF II, NIDEK, Tokyo, Japan); and subjective refraction using a phoropter (KR-800, Topcon, Tokyo, Japan). For children and adolescents under 12 years of age, cycloplegic refraction was conducted with compound tropicamide eye drops (brand name: ZHUOBIAN^®^, Shenyang Xingqi Pharmaceutical Co., Ltd., Shenyang, China), instill one drop every five minutes for a total of three doses. Refraction should be performed 20 min after the last instillation, Perform adequate fogging prior to refraction in individuals aged 12 years or older to minimize accommodative influence. Axial length (AL) was measured using an optical biometer (SW-9000, Suowei, Tianjin, China). Anterior segment examination was performed with a slit-lamp biomicroscope (LS-7, Shangbang, Chongqing, China). Fundus imaging was conducted using a non-mydriatic ultra-widefield scanning laser ophthalmoscope (Optos, Nikon, Tokyo, Japan) to obtain 200° to 240° field-of-view images (Figure 1).

### 2.3. Standardization and Quality Control

Examinations followed a unified, standardized operational protocol using equipment of identical specifications to ensure data consistency and comparability. A fixed, dedicated team performed the examinations, comprising a licensed ophthalmologist and several professionals certified as optometrists or with equivalent qualifications. All operators underwent systematic training to master the standardized operating procedures and examination techniques. They were required to pass both theoretical and practical assessments, confirming proficiency before participating in the actual examinations. This rigorous process was implemented to minimize inter-operator variability and ensure the reliability and reproducibility of the collected data.

The diagnosis of LD was established primarily using ultra-widefield scanning laser ophthalmoscopy. The condition is characterized by: (1) well-circumscribed, oval or band-shaped atrophic areas in the peripheral retina; (2) a distinctive lattice pattern of interlacing white linings within the lesions; and (3) frequent concomitant perilesional pigmentary disturbances. In atypical presentations, red-green dual-channel imaging was employed for a detailed assessment of the retinal and choroidal architecture, thereby aiding definitive diagnosis.

### 2.4. Data Analysis

To maintain statistical independence for primary analyses, one eye per participant was analyzed (N = 1776). Affected eyes were prioritized for unilateral cases; otherwise, selection was random.

Statistical analyses were performed using R software (version 4.5.1). The normality of continuous variables was assessed using the Kolmogorov–Smirnov test. When comparing baseline characteristics between healthy participants and patients with LD, variables conforming to a normal distribution were evaluated for homogeneity of variance using Levene’s test. If variances were equal, an independent samples t-test was applied; if variances were unequal, the Welch t-test was used. For variables that did not follow a normal distribution, the Mann–Whitney U test was employed for comparison. Differences in gender composition between groups were analyzed using the chi-square test. The chi-square test was also used to examine differences in the occurrence of LD across different patient subgroups.

Correlation and logistic regression analyses were conducted to evaluate the associations of patient-level and ocular indicators with LD. Pearson’s correlation coefficient was used for normally distributed continuous variables. Spearman’s rank correlation coefficient was applied when at least one variable was non-normally distributed or binary. The phi coefficient was calculated for associations between two binary variables. Univariate and multivariate logistic regression models were subsequently constructed to further analyze the strength of the association between each factor and LD.

Normally distributed continuous data are presented as mean ± standard deviation. Non-normally distributed data are reported as median (first quartile, third quartile). All statistical tests were two-sided, with a significance level (α) set at 0.05.

### 2.5. Definitions of Key Variables

In this study, participants were categorized as myopic if their spherical equivalent (SE) was ≤−0.50 diopters (D). Conversely, participants with an SE > −0.50 D were categorized as non-myopic [23].

## 3. Results

### 3.1. Participant Demographics and Ocular Characteristics

Demographic Characteristics: A total of 1776 participants (773 males, 1003 females), ranging in age from 3 to 80 years, were enrolled in this study. The demographic characteristics of the participants are summarized in Table 1.

Ocular Characteristics and Statistical Analysis: A total of 1772 eyes from the enrolled participants were included. The baseline ocular characteristics and comparative results are presented in Table 2. No statistically significant differences were observed between groups in ocular laterality or IOP (all *p* > 0.05). However, significant differences were noted in AL, SE, and Km (all *p* < 0.05).

### 3.2. Comparison of Prevalence Rates Between Different Groups

Based on an analysis of 1776 patients, the overall prevalence of LD was found to be 13.3%. The prevalence among minors was 5.9%. Of these 237 patients, 166 (70.0%) had unilateral involvement and 71 (30.0%) had bilateral involvement (Table 3). The proportion of unilateral LD was significantly higher than that of bilateral LD (*p* < 0.001). Furthermore, the prevalence of LD in the myopic eyes was 15.5%, which was significantly higher than the 10.5% observed in the non-myopic eyes (*p* < 0.001). Regarding sex, the prevalence rates were 13.0% in males and 13.7% in females, with no statistically significant difference between the groups. Additionally, no significant difference in prevalence was found concerning dominant eye status (*p* > 0.05).

### 3.3. Correlation Analysis of SE, AL, AL/CR, and Age with LD

**Figure 2:**
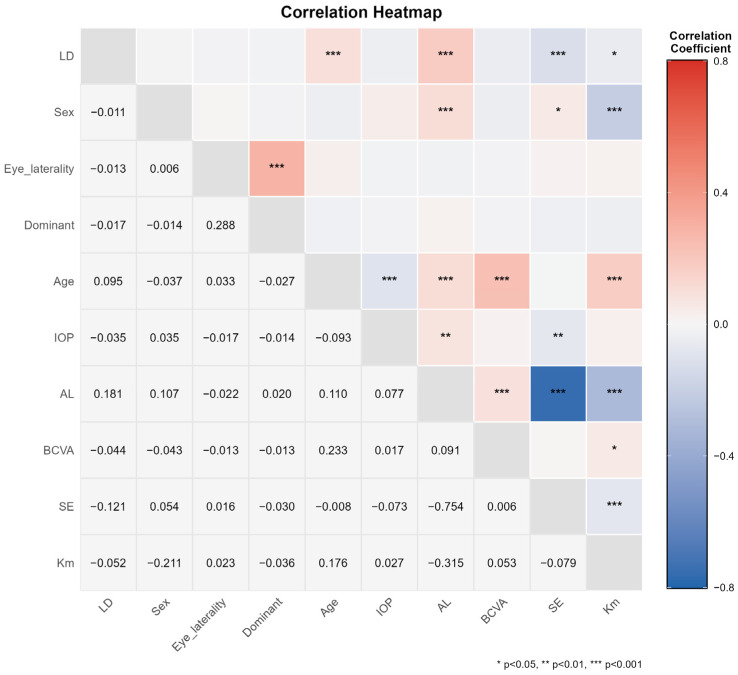
Heatmap Analysis of Correlations Between Demographic Characteristics, Ocular Biometric Parameters, and Lattice Degeneration. BCVA, which stands for best-corrected visual acuity, has been converted to logarithmic Minimum Angle of Resolution (logMAR) visual acuity. LD, lattice degeneration; IOP, intraocular pressure; AL, axial length; SE, spherical equivalent; Km, mean keratometry.

Spearman’s rank correlation analysis was performed to evaluate the associations of SE, AL and age with LD (Figure 2). The analysis revealed a weak positive correlation between the absolute value of SE and LD (Spearman’s *ρ* = −0.121, *p* < 0.001). A significant positive correlation was observed between AL and LD (*ρ* = 0.18, *p* < 0.001), indicating that the risk of LD increases with greater AL. A significant positive correlation was also found between AL/CR and LD (*ρ* = 0.14, *p* < 0.001). Although the correlation between age and LD was relatively weak (*ρ* = 0.10, *p* < 0.001), it remained statistically significant, suggesting a slight increase in LD risk with advancing age.

In the large sample of 1776 eyes, the strength of association between each variable and LD, in descending order, was as follows: AL (*ρ* = 0.18, *p* < 0.001) > SE (*ρ* = −0.12, *p* < 0.001) > age (*ρ* = 0.10, *p* < 0.001). Furthermore, Km (*ρ* = −0.05, *p* = 0.029) also demonstrated a statistically significant, albeit weak, correlation with LD.

AL showed significant correlations with other ocular parameters: a strong negative correlation with SE (*ρ* = −0.75, *p* < 0.001), consistent with the biological progression of myopia; a negative correlation with Km (*ρ* = −0.32, *p* < 0.001); and a positive correlation with IOP (*ρ* = 0.08, *p* = 0.001). Analysis of sex-related factors indicated that female sex was associated with steeper corneal curvature (*ρ* = −0.21, *p* < 0.001) and positively correlated with greater AL (*ρ* = 0.11, *p* < 0.001). Age was positively correlated with worse BCVA (*ρ* = 0.23, *p* < 0.001), suggesting a potential decline in visual function with increasing age.

### 3.4. Univariate Logistic Regression Analysis

Univariate logistic regression models were constructed to further quantify the influence of each factor on LD. For the absolute value of SE, the regression coefficient β was 0.08 (OR = 1.08, 95% CI: 1.05–1.12, *p* < 0.001), indicating that for each 1 D increase in the absolute SE, the risk of LD increased by approximately 8%. For AL, β was 0.27 (OR = 1.31, 95% CI: 1.22–1.41, *p* < 0.001), meaning that each 1 mm increase in AL was associated with a 31% increase in LD risk. For AL/CR, β was 1.65 (OR = 5.22, 95% CI: 2.91–9.34, *p* < 0.001), suggesting that each unit increase in AL/CR raised the LD risk by approximately 422%, identifying it as a strong risk factor for LD. Based on this model, the predicted probability of LD was 10.4%, 12.0%, and 15.2% at AL/CR values of 3.01, 3.10, and 3.27, respectively. Detailed data are presented in Table 4.

**Figure 3:**
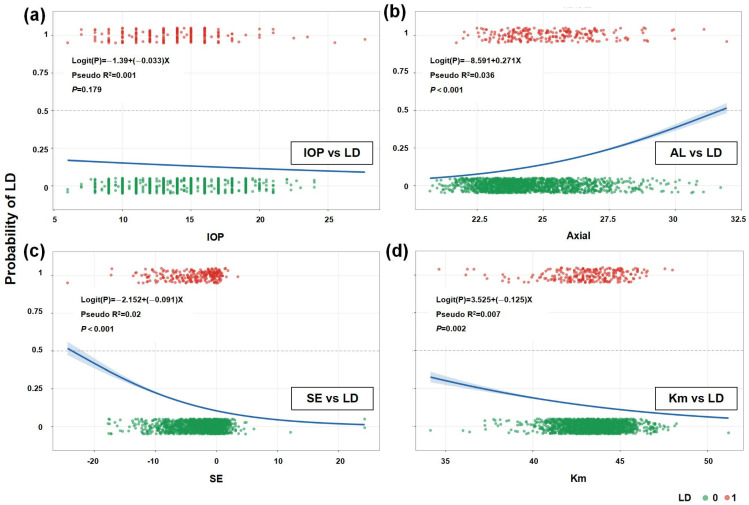
Logistic Regression Scatter Plot. Panels (**a**–**d**) represent the correlation analysis of IOP, AL, SE, and Km with LD, respectively.

Visualization of the logistic regression analysis indicated no significant association between IOP and LD (Figure 3a), suggesting limited predictive value of IOP for LD risk. In contrast, AL showed a significant positive correlation with LD (Figure 3b), providing a quantitative basis for using AL in clinical risk assessment for LD. SE was negatively correlated with LD (Figure 3c), indicating a significantly increased risk of LD in patients with high myopia. Km also showed a negative correlation with LD (Figure 3d), although the association was weak, suggesting that corneal curvature may serve as a supplementary rather than a primary predictive indicator.

### 3.5. Multivariate Logistic Regression Analysis

**Figure 4:**
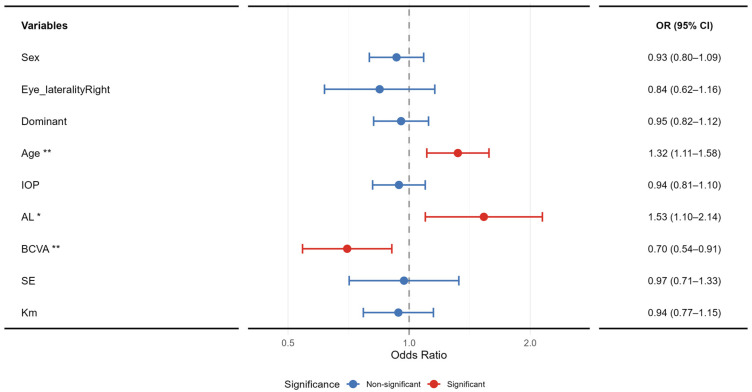
Results of multivariate logistic regression analysis of risk factors for lattice degeneration. The figure shows the adjusted odds ratios and 95% CIs for all candidate factors. Red lines indicate a statistically significant association, while blue lines indicate a statistically non-significant association. Among them, OR represents the odds ratio, and CI represents the confidence interval. * *p* < 0.05. ** *p* < 0.001.

Multivariable logistic regression analysis was employed to systematically examine the relationship between multiple factors [24]—including sex, age, IOP, AL, and BCVA—and the risk of LD (Figure 4). This approach aimed to enhance the model’s goodness-of-fit and predictive accuracy. The regression model was constructed by incorporating variables such as sex, laterality (eye), dominant eye, age, IOP, AL, BCVA, SE, and Km to identify independent factors influencing LD. The model’s goodness-of-fit was assessed using Adjusted R^2^, Akaike’s Information Criterion (AIC), and the Bayesian Information Criterion (BIC). A significance level of *p* < 0.05 was set for all variables.

The multivariable logistic regression model demonstrated a modest fit, with McFadden R^2^ = 0.05 and Nagelkerke R^2^ = 0.07. The Akaike and Bayesian Information Criteria were 1185.16 and 1238.90, respectively. The likelihood ratio test for the overall model was statistically significant (*p* < 0.001), confirming that the model as a whole was meaningful. The analysis identified age (OR = 1.32, 95% CI: 1.11–1.58, *p* = 0.002), AL (OR = 1.53, 95% CI: 1.10–2.14, *p* = 0.012), and BCVA (OR = 0.70, 95% CI: 0.54–0.91, *p* = 0.007) as independent factors significantly associated with lattice degeneration (LD). Other variables, including sex, eye laterality, dominant eye, intraocular pressure, spherical equivalent, and mean keratometry, were not significantly associated with LD in the final model (all *p* > 0.05). Detailed results are presented in Table 5.

## 4. Discussion

LD is an important precursor lesion of rhegmatogenous retinal detachment, with literature reports of its prevalence in general populations typically ranging from 4.5% to 10% [3,5,25,26]. However, our large-scale screening revealed a higher overall prevalence of 13.3%. This elevated rate, which notably exceeds the upper limit of previously reported estimates, may be related to advancements in examination technology (e.g., the use of ultra-widefield imaging) or distinct demographic characteristics of the enrolled population.

While previous studies have indicated a relatively balanced distribution between unilateral and bilateral LD (54.2% vs. 45.8%) [23], Our results revealed a significantly higher prevalence of unilateral LD compared to bilateral involvement (see Table 3). This suggests that the occurrence of LD may be influenced more by localized, eye-specific factors rather than systemic predispositions. This interpretation aligns more closely with the current understanding of LD’s core pathogenesis, which emphasizes local degeneration and traction [6].

Regarding age distribution, our results support the overall trend of increasing LD prevalence with advancing age (see Figure 2) [27]. Although detailed assessment of the peripheral retina is often overlooked in routine eye examinations for children and adolescents, a prevalence of 5.9% was still observed in this population. Therefore, systematic peripheral retinal examinations are warranted in minors. Furthermore, no significant difference in LD prevalence was found between dominant and non-dominant eyes (11.9% vs. 13.0%) (see Table 3), suggesting that ocular dominance is not associated with the risk of developing LD.

The results in Table 3 confirm that myopia is a significant risk factor for LD, The prevalence of LD was significantly higher in myopic individuals (15.5%) than in non-myopic individuals (10.5%). However, direct comparisons of absolute prevalence rates across studies are complicated by methodological heterogeneity, including variations in study design, participant selection, and diagnostic criteria. This lack of standardization is reflected in the broad range of myopia prevalence reported in prior population-based studies, which spans from approximately 5.8% to 33% [14,28] and frequently clusters around 12–15% [11,14,29,30]. Therefore, the methodological context is essential for the accurate interpretation and cross-study comparison of such epidemiological findings.

Further multivariate analysis demonstrated that SE was not an independent risk factor for LD (See Figure 4). Although a weak positive correlation was observed in univariate assessment, its standalone predictive value is limited. In contrast, axial length (AL) demonstrated a robust and independent association with lattice degeneration (LD) in both univariate and multivariate analyses. Univariate logistic regression indicated that each 1 mm increase in AL was associated with a 31% increase in LD risk (OR = 1.31, 95% CI: 1.22–1.41, *p* < 0.001). This finding was further supported by multivariate analysis, which revealed an even stronger independent effect (OR = 1.53, 95% CI: 1.10–2.14, *p* = 0.012); after adjusting for other factors, each 1 mm increase in AL raised the odds of LD by approximately 53%.

The axial length to AL/CR, a composite metric often regarded as superior to AL alone for myopia assessment and a focus of recent research [31], was therefore also analyzed. However, the correlation between LD and AL (*ρ* = 0.18) proved stronger than that between LD and AL/CR (*ρ* = 0.14), and AL/CR was not identified as an independent risk factor for LD in the multivariate model. These results suggest that although AL/CR is a valuable composite measure of the eye’s overall optical configuration, LD may be more directly driven by excessive axial elongation, which induces mechanical stretching and thinning of the retina and vitreous [6], ultimately compromising blood supply and leading to degenerative changes [5].

The optimal management strategy for LD remains a subject of clinical discussion. Current guidelines advocate a conservative approach, generally advising against prophylactic laser photocoagulation for asymptomatic lesions without high-risk features [2]. Nevertheless, an alternative perspective is offered by Tsai et al. [2,32], who posit that retinopexy may confer benefits by alleviating post-intervention vitreoretinal traction, suggesting that early preventive laser therapy deserves careful consideration in select cases. Given that LD-related retinal detachment can lead to severe visual impairment, modern clinical decision-making should be based on precise evaluation utilizing advanced imaging technologies like ultra-widefield fundus imaging and OCT. Treatment or follow-up plans should be individualized, carefully weighing the potential benefits of prophylactic treatment against its risks.

This study employed a two-stage analytical strategy, progressing from univariate screening to multivariable adjustment. While univariate analysis identified several correlates of LD, the key advantage of the multivariable logistic regression was its identification of age, AL, and BCVA as independent risk factors after controlling for potential confounders (see Figure 4).The model’s relatively low explanatory power (Nagelkerke R^2^ = 0.07), however, indicates the material influence of numerous unmeasured variables—consistent with LD’s complex, multifactorial etiology involving genetics, vitreoretinal interface microstructure, and local blood supply—which limits its utility for individual-level prediction. Furthermore, this study has several inherent limitations. First, its cross-sectional design precludes the inference of causality. Second, the single-center cohort may constrain the generalizability of the findings. Third, the lack of genetic and family history data impedes the exploration of potential hereditary mechanisms. Finally, as all diagnoses were made by a single physician, inter-rater reliability (e.g., via Kappa statistics) could not be assessed, which may introduce diagnostic consistency bias. Notwithstanding these limitations, our systematic analysis provides robust cross-sectional evidence that helps to delineate key risk factors, laying a necessary foundation for constructing a clinical risk assessment framework. The ultimate goal of such a framework is to accurately stratify individuals and identify those at genuinely high risk, thereby enabling targeted monitoring or early intervention to improve patient outcomes. Future prospective studies that integrate multi-omics data and advanced imaging phenotypes will be essential to refine this framework and establish causal pathways.

## 5. Conclusions

In summary, this study demonstrates that LD has an overall prevalence of 13.3% in the studied population, occurs predominantly unilaterally, and is closely associated with myopia, particularly AL elongation. AL was identified as a stronger predictive indicator for LD risk than the AL/CR ratio, supporting the central role of mechanical traction in its pathogenesis. Although some LD cases progress slowly, early evaluation using modern imaging techniques like ultra-widefield imaging and individualized management for cases with high-risk features are recommended to mitigate the risk of retinal detachment.

## Figures and Tables

**Table 1:** Demographic Characteristics of the Study Participants (N = 1776).

Parameters	Overall Distribution	Non-LD	LD	*t/χ^2^*	*p*
Age, years	33.23 ± 21.12	32.58 ± 21.64	37.39 ± 16.88	−3.9136	0.0001 *
Sex (n%)				0.1395	0.7088
Male	773 (43.5)	673 (43.6)	100 (42.2)		
Female	1003 (56.5)	866 (56.4)	137 (57.8)		

Data are presented as Mean ± standard deviation (SD) for measurement data and as frequency for enumeration data. Comparisons between groups for categorical variables were performed using the chi-square test, with χ^2^ values reported; normally distributed continuous variables were analyzed using the independent samples t-test, with t values reported. LD, lattice degeneration. * *p* < 0.05.

**Table 2:** Baseline Ocular Characteristics and Intergroup Comparisons (N = 1776).

Parameters	Overall Distribution	Non-LD (n = 1539)	LD (n = 237)	*t/χ^2^/U*	*p*
Eye Lateral (R/L)	862/914	751/788	111/126	0.2430	0.6221
IOP (mmHg)	13.81 ± 2.9	13.85 ± 2.85	13.55 ± 3.16	−1.2469	0.2134
AL (mm)	24.51 ± 1.71	24.39 ± 1.68	25.27 ± 1.74	7.2989	<0.001 *
SE (d) ^#^	−1.5 [−4.38, −0.12]	−1.38 [−4.12, 0]	−2.88 [−6.50, −0.5]	144,863.0	<0.001 *
Km (d)	43.42 ± 1.67	43.46 ± 1.6	43.10 ± 2.03	−2.6311	0.009 *

# SE did not follow a normal distribution and is presented as median [interquartile range]. Non-normally distributed continuous variables were analyzed using the Mann–Whitney U test, with the U statistic reported. R/L, right eye/left eye; IOP, intraocular pressure; AL, axial length; SE, spherical equivalent; Km, mean keratometry. * *p* < 0.05.

**Table 3:** Comparison of Prevalence Rates Between Different Groups.

Comparison Group	Number of Disease Cases	Total (N)	Prevalence (%)	*p*	Test Statistic (χ^2^)
Monocular vs. Binocular	166/71	1776	9.3/4.0	<0.001 *	38.08
Dominant Eye vs. Non-dominant Eye	97/109	1656	11.9/13.0	0.538	0.38
Myopic vs. non-myopic	162/74	1776	15.5/10.5	0.004 *	8.47
Male vs. Female	100/137	1776	13.0/13.7	0.709	0.14

The methodological foundation for analyzing unilateral and bilateral prevalence was at the individual level, whereas all other analyses were conducted at the eye level. Data for “Dominant vs. Non-dominant Eye” included 1656 participants due to incomplete documentation of ocular dominance. * *p* < 0.05.

**Table 4:** Logistic Regression Analysis of the Relationship between Ocular Biometry and LD.

Parameters	β	OR	95% CI of OR		*p*	AIC
			Lower	Upper		
SE	0.08	1.08	1.05	1.12	<0.001 *	1379.8
AL	0.27	1.31	1.22	1.41	<0.001 *	1349.3
AL/CR	1.65	5.22	2.91	9.34	<0.001 *	1369.7

β, Regression Coefficient; OR, Odds Ratio; SE, Spherical Equivalent; AL, Axial Length; AL/CR, Axial Length/Corneal Radius Ratio. AIC, Akaike Information Criterion; CI, Confidence Interval. The Hosmer–Lemeshow goodness-of-fit test indicated adequate fit for all models (all *p* > 0.05). The area under the receiver operating characteristic curve (AUC) was 0.58 for the SE model, 0.65 for the AL model, and 0.62 for the AL/CR model. * *p* < 0.05.

**Table 5:** Multivariable Logistic Regression Analysis for Lattice Degeneration.

Parameters	OR	95% Cl	*p* Value
Sex	0.93	0.80–1.09	0.362
Eye (Right)	0.85	0.62–1.16	0.295
Dominant Eye	0.96	0.82–1.12	0.565
Age	1.32	1.11–1.58	0.002 *
IOP	0.94	0.81–1.10	0.448
AL	1.53	1.10–2.14	0.012 *
BCVA	0.70	0.54–0.91	0.007 *
SE	0.97	0.71–1.33	0.854
Km	0.94	0.77–1.15	0.547

* *p* < 0.05.

## Data Availability

The data presented in this study are available on request from the corresponding author.

## Abbreviations

The following abbreviations are used in this manuscript:

LD	Lattice Degeneration
SE	Spherical Equivalent
AL	Axial Length
AL/CR	Axial Length/Corneal Radius Ratio
BCVA	Best-Corrected Visual Acuity
OCT	Optical Coherence Tomography
RD	Retinal Detachment
Km	Mean Keratometry
IOP	Intraocular Pressure
AIC	Akaike’s Information Criterion
BIC	Bayesian Information Criterion
OR	Odds Ratio
CI	Confidence Interval
logMAR	logarithmic Minimum Angle of Resolution
